# Single nucleotide polymorphism rs13042395 in the *SLC52A3* gene as a biomarker for regional lymph node metastasis and relapse-free survival of esophageal squamous cell carcinoma patients

**DOI:** 10.1186/s12885-016-2588-3

**Published:** 2016-07-29

**Authors:** Hua-Zhen Tan, Zhi-Yong Wu, Jian-Yi Wu, Lin Long, Ji-Wei Jiao, Yu-Hui Peng, Yi-Wei Xu, Shan-Shan Li, Wei Wang, Jian-Jun Zhang, En-Min Li, Li-Yan Xu

**Affiliations:** 1 Key Laboratory of Molecular Biology in High Cancer Incidence Coastal Chaoshan Area of Guangdong Higher Education Institutes, Shantou University Medical College, No. 22, Xinling Road, Shantou, 515041 China; 2 Department of Biochemistry and Molecular Biology, Shantou University Medical College, No. 22, Xinling Road, Shantou, 515041 China; 3 Department of Oncologic Surgery, Shantou Central Hospital, Affiliated Shantou Hospital of Sun Yat-Sen University, Shantou, 515041 China; 4 Department of Clinical Laboratory, Cancer Hospital of Shantou University Medical College, No.7, Raoping Road, Shantou, Guangdong 515041 China; 5 Department of Preventive Medicine, Shantou University Medical College, No. 22, Xinling Road, Shantou, 515041 China; 6 Institute of Oncologic Pathology, Shantou University Medical College, No. 22, Xinling Road, Shantou, 515041 China

**Keywords:** Esophageal squamous cell carcinoma, Single nucleotide polymorphism, *SLC52A3* gene, Tumor characteristics, Relapse-free survival

## Abstract

**Background:**

*SLC52A3* was recently identified as a susceptibility gene for esophageal squamous cell carcinoma (ESCC). However, associations between the single nucleotide polymorphisms (SNPs) rs13042395 (C > T) and rs3746803 (G > A) in *SLC52A3* and risk, tumor characteristics and survival of ESCC patients remain inconclusive and of unknown prognostic significance.

**Methods:**

Analyses of the association between SNPs in *SLC52A3* and ESCC risk were performed on 479 ESCC cases, together with 479 controls, in a case-control study. Blood samples for cases and controls were collected and genotyped by real-time polymerase chain reaction (PCR) using TaqMan assays. Among the 479 ESCC cases, 343 cases with complete clinical data were used to investigate the association between SNPs and ESCC clinical characteristics; 288 cases with complete clinical data and 5-year follow-up data were used to analyze the association between SNPs and prognosis. Dual luciferase reporter assays and electrophoretic mobility shift assays (EMSAs) were used to investigate the biological function of rs13042395.

**Results:**

No association was found between *SLC52A3* rs3746803 and susceptibility, tumor characteristics or survival of ESCC patients. For rs13042395, TT genotype carriers were likely to have reduced lymph node metastasis (odds ratio (OR) = 0.55, 95 % confidence interval (CI), 0.31–0.98) and longer relapse-free survival time (*P* = 0.03) . Also, both rs13042395 (hazard ratio (HR) = 0.62, 95 % CI, 0.38–0.99) and regional lymph node metastasis (HR = 2.06, 95 % CI, 1.36–3.13 for N1 *vs*. N0; HR = 2.88, 95 % CI, 1.70–4.86 for N2 *vs*. N0; HR = 2.08, 95 % CI, 1.01–4.30 for N3 *vs*. N0) were independent factors affecting relapse-free survival for ESCC patients who underwent surgery. Dual luciferase reporter assays and EMSAs suggested that the CC genotype of rs13042395 enhanced *SLC52A3* expression, probably via binding with specific transcription factors.

**Conclusions:**

The rs13042395 polymorphism in *SLC52A3* is associated with regional lymph node metastasis and relapse-free survival in ESCC patients.

**Electronic supplementary material:**

The online version of this article (doi:10.1186/s12885-016-2588-3) contains supplementary material, which is available to authorized users.

## Background

Esophageal cancer (EC) is the tenth most common cancer worldwide [1]. According to a Chinese national annual cancer registration report in 2010, esophageal cancer is the fifth most common malignant tumor in China, with an incidence of 21.88/10^5^ [2]. EC has two main histologic subtypes: esophageal squamous cell carcinoma (ESCC) and esophageal adenocarcinoma (EAC). ESCC has a distinct geographic distribution worldwide with higher prevalence in central Asia and southern Africa, and accounts for about 90 % of all EC cases in China [3]. The survival for ESCC patients is poor, with a 5-year overall survival rate below 13.0 % [4, 5]. On one hand, this outcome is partly because of the lack of effective biomarkers for the early detection of ESCC, which results in most ESCC cases presenting at an advanced stage at the time of diagnosis [6]. On the other hand, due to a lack of early warning biomarkers for relapse after surgery, ESCC is difficult to prevent and control relapse and prolong relapse-free survival. Therefore, effective biomarkers for the early detection and relapse of ESCC are urgently needed.

Single nucleotide polymorphisms (SNPs) are regarded as stable and effective biomarkers for prediction of onset and susceptibility, and prognosis of various cancers. In recent years, genome-wide association studies (GWAS) of ESCC in Chinese populations indicate that SNP loci in the *PLCE1*, *CASP8*, *TMEM173*, *ATP1B2* and *SLC52A3* genes are associated with ESCC susceptibility [7–11]. *SLC52A3* (also named *C20orf54*) on chromosome 20p13 encodes human riboflavin transporter 2 (RFT2), a trans-membrane protein that specifically and efficiently transports riboflavin into cells, playing a role in riboflavin homeostasis [12, 13]. More importantly, it has been reported that *SLC52A3* is frequently overexpressed in tumors, compared with normal adjacent tissue, in ESCC patients. Knockdown of *SLC52A3* in ESCC cells results in inhibition of cell proliferation, colony formation and anchorage-independent growth, whereas overexpression of *SLC52A3* in ESCC cells promotes cell proliferation, confers resistance to cisplatin and enhances tumorigenicity in nude mice [14]. All these indicate that *SLC52A3* plays an important role in ESCC tumorigenesis and prognosis. A recent GWAS study and smaller studies have shown that some SNP loci, such as rs13042395 (C > T), rs3746802 (T > C), rs3746803 (G > A) and rs3746804 (G > A) in *SLC52A3*, are associated with ESCC risk [8, 15, 16]. The rs13042395 is a SNP locus, located at the 5′ flanking region of the *SLC52A3* gene, with a minor allele frequency (MAF) ranging from 9.30 to 36.4 % in ESCC *vs*. 8.28 % to 36.5 % in controls [8, 9, 17–20]. However, according to other GWAS studies and smaller sample replication studies, associations between rs13042395 and ESCC are inconclusive [9, 11, 17–20]. The rs3746803, located in the coding region of *SLC52A3*, is a functional polymorphism (missense) and site of modification by protein kinase C. The reported MAF of rs3746803 in the PubMed SNP database is 9.05 %. So far, only one study demonstrates that no relationship exists between rs3746803 and risk of ESCC [15]. Moreover, neither rs13042395 nor rs3746803 have been validated in regard to whether they are related to ESCC in the Chaoshan area of China, a coastal high-risk area for EC, and it has yet to be reported whether SNPs rs13042395 and rs3746803 are associated with tumor characteristics and survival in ESCC patients.

In the present study, we investigated the association between rs13042395 or rs3746803, in *SLC52A3*, and ESCC risk, tumor characteristics and survival. We used a large Chinese study population (in the Chaoshan area) that has detailed clinical data and a follow-up time of 5 years. SNPs were genotyped by the Taqman polymerase chain reaction (PCR) method, and luciferase reporter assays and electrophoretic mobility shift assays (EMSAs) were conducted to explore how the SNPs regulate *SLC52A3* expression.

## Methods

### Study population

Study participants for the present study were drawn from the Chaoshan region in China (a coastal high-risk area for ESCC). Analyses of the association between *SLC52A3* SNPs and ESCC risk were performed on 479 ESCC cases together with 479 controls. All ESCC cases were diagnosed histopathologically. The controls were matched by gender and age, and were selected from healthy persons who had physical examinations in Shantou Central Hospital (cancer patients were excluded). Blood samples for cases and controls were collected between January 2008 and January 2014. The volume of blood samples of all ESCC cases and controls was more than 3 milliliters. Three hundred forty-three, of the 479 ESCC cases, were used to analyze association between SNPs and ESCC tumor characteristics because they had undergone surgery and had detailed clinical data (Table 1). Clinical data for ESCC cases was retrieved from Shantou Central Hospital and the Cancer Hospital of Shantou University Medical College. Two hundred eighty-eight of the 343 cases participated in follow-up studies performed from the 1st of January, 2008, until the 31st of December 2014. Information about the date of death and relapse after surgery was collected. Detailed clinical data and follow-up data of the 288 ESCC cases were used to analyze the association between SNPs and survival of ESCC patients. All participants in the present study have signed informed consent. This study was approved by the Ethics Committee of Shantou University Medical College.

**Table 1:** Characteristics of ESCC cases and controls

Variables	SNP and ESCC risk		SNP and ESCC progression	SNP and ESCC prognosis
	ESCC (*N* = 479)	Control (*N* = 479)	ESCC (*N* = 343)	ESCC (*N* = 288)
Gender				
Female	109	109	87	79
Male	370	370	256	209
Age (years) (M ± SD)	59.92 ± 9.34	59.61 ± 8.84	58.39 ± 8.83	58.28 ± 8.84
Size of tumor (cm)				
≤3	101		98	89
4–5	160		158	130
≥6	89		87	69
Unknown	129		0	0
Depth of tumor invasion				
Tis	8		8	5
T1	29		29	24
T2	45		45	39
T3	239		239	216
T4	22		22	4
Unknown	136		0	0
Regional lymph node metastasis				
N0	178		178	150
N1	103		103	88
N2	40		40	34
N3	22		22	16
Unknown	136		0	0
Distant metastasis				
M0	337		337	288
M1	6		6	0
Unknown	136		0	0
Tumor location				
Upper thoracic	56		27	19
Middle thoracic	251		191	158
Lower thoracic	143		125	111
Unknown	29		0	0
TNM classification				
0	8		8	4
I	42 (3)^a^		39	35
II	156 (18)^a^		138	124
III	185 (33)^a^		152	125
IV	20 (14)^a^		6	0
Unknown	68		0	0
Radiotherapy after surgery				
No			204	204
Yes			84	84
Unknown			55	0
Chemotherapy after surgery				
No			207	207
Yes			81	81
Unknown			55	0

All associations are significant at *P* < 0.05

*ESCC* esophageal squamous cell carcinoma, *SNP* single nucleotide polymorphism, *M* mean, *SD* standard deviation

^a^Data shown in parentheses represent patients without tumor resection

### DNA extraction and SNP genotyping

Genomic DNA was extracted from whole blood with a TIANamp Blood DNA Kit (TIANGEN BIOTECH, Beijing, China). Genotyping was performed using a TaqMan PCR allelic discrimination method with an ABI 7500 Real-Time PCR System (Applied Biosystems, Foster City, California, USA). Predesigned TaqMan SNP genotyping assays were used, with minor groove binding probes 5′-labelled with VIC or FAM fluorophores (Applied Biosystems). PCR was performed with 20 ng genomic DNA in a total reaction volume of 5 μl, using 40× Taqman SNP Genotyping Assay (Applied Biosystems), 2× Taqman Genotyping Master Mix (Applied Biosystems) and water. PCR was performed under the following conditions: an initial holding at 95 °C for 10 min, 40 cycles of denaturation at 92 °C for 15 s and annealing and extension at 60 °C for 1 min, and a final holding at 60 °C for 1 min. All blood samples were genotyped successfully.

### Assembly of reporter constructs

We prepared a 400 bp genomic DNA fragment containing the human rs13042395 locus located 5622 nt upstream of the transcriptional starting site in the human *SLC52A3* gene. The fragment was generated by PCR with a forward primer containing a *Kpn*I site (underlined) 5′-GGTACCTAATGCGTGGGCGACAGA-3′ and a reverse primer containing an *Xho*I site (underlined) 5′-CTCGAGGTGGCAAGCCAGATGGT-3′ and inserted into the pGL3-Promoter (pGLP) reporter vector (Promega, Madison, WI, USA) to create the pGLP-C construct, in which the SNP locus contained the C allele. The pGLP-C construct then underwent site-directed mutagenesis to engineer the pGLP-T construct in which the SNP locus contained the T allele. Mutagenesis was conducted by PCR (primers: 5′-CAGGGCCAGTGCACCGTTATTGTGTGGGCTGGG-3′; 5′-AACGGTGCACTGGCCCTGGTCAGAACCCCACTC-3′) using the Fast Mutagenesis System (TransGen Biotech, Beijing, China).

### Cell culture and dual luciferase reporter assay

The human KYSE150 and KYSE180 esophageal squamous carcinoma cell lines were cultured in RPMI-1640 medium (ThermoFisher, HyClone, CA, USA) supplemented with 10 % fetal bovine serum (Life Technologies, Australia). All cells were maintained at 37 °C in a humidified 5 % CO_2_ atmosphere.

Cells were inoculated in 96-well plates at 1.5 × 10^5^ cells/ml, grown to 50–80 % confluence and co-transfected with 0.5 μg of either pGLP-C or pGLP-T, and 0.01 μg control vector (*Renilla* luciferase plasmid pRL-TK (Promega, Madison, WI, USA)), using Superfect Transfection Reagent (QIAGEN, Hilden, Germany) according to the manufacturer’s instructions. The experimental reporter vector contained a modified coding region for *firefly* (*Photinuspyralis*) luciferase that has been optimized for monitoring transcriptional activity in transfected eukaryotic cells. After transfection, cells were incubated for 48 h and harvested in Passive Lysis Buffer (Promega, Madison, WI, USA). The luciferase reporter activity of the lysates was measured using the Dual-Luciferase Reporter Assay System (Promega, Madison, WI, USA) according to the manufacturer’s recommendations.

### Electrophoretic mobility shift assay (EMSA)

Nuclear extracts from KYSE150 cells were prepared using NE-PER Nuclear and Cytoplasmic Extraction Reagent Kit (Thermo Pierce Biotechnology, Rockford, IL, USA) according to the manufacturer’s instructions. EMSA was performed using biotin 3′-end labelled 30 bp probes for the rs13042395 locus. Equimolar amounts of complementary and single-stranded oligonucleotides were annealed. The oligonucleotide probes used in EMSAs were 5′- GGCCAGTGCACCGTCATTGTGTGGGCTGGG-3′ and 5′-CCCAGCCCACACAATGACGGTGCACTGGCC-3′ for the CC genotype; and 5′-GGCCAGTGCACCGTTATTGTGTGGGCTGGG-3′ and 5′-CCCAGCCCACACAATAACGGTGCACTGGCC-3′ for TT genotype, in which underlined nucleotides indicate the SNP locus. In specific competition experiments, a 200-fold molar excess of unlabeled oligonucleotides was added to the binding reaction. Probes were incubated with 3 μg of nuclear protein extracts for 25 min at room temperature. The remaining steps followed the Light Shift Chemiluminescent EMSA Kit protocol (Thermo Pierce Biotechnology, Rockford, IL, USA).

### Statistical analysis

The observed genotype frequencies in the controls were tested for Hardy–Weinberg equilibrium using free online software (http://analysis.bio-x.cn/myAnalysis.php) [21]. Odds ratios (ORs) and 95 % confidence intervals (95 % CIs) for association between the *SLC52A3* SNPs and ESCC risk were calculated by binary logistic regression. Ordinal logistic regression was performed to estimate OR and 95 % CI for association between SNPs and ESCC characteristics. Survival curves for ESCC relapse-free survival and overall survival after surgery were derived by the Kaplan–Meier method. Univariate Cox regression was used to estimate hazard ratio (HR) and 95 % CI for risk factors related to ESCC relapse-free survival and overall survival, respectively. Furthermore, in order to determine the value of certain risk factors as independent prognostic factors, multivariate Cox regression was performed to analyze the HR of the overall risk factors (adjusted for each other) for ESCC relapse-free survival and overall survival. TMN stage was composed of depth of tumor invasion (T), regional lymph node metastasis (N) and distant metastasis (M). There were collinearities between TNM stage and tumor invasion, regional lymph node metastasis and distant metastasis, respectively. In order to get more information about the relation between tumor invasion, regional lymph node metastasis, distant metastasis and survival, we excluded TNM stage, in the multivariate Cox analysis, by a forward stepwise method. Relative luciferase activity was defined as *firefly* luciferase activity per *Renilla* luciferase activity in transfected cells. One-way analysis of variance along with the Bonferroni post hoc test was used to determine whether differences were significant for relative luciferase activity between groups. All analyses mentioned above were performed using SPSS, version 16.0 software (IBM SPSS, Chicago, IL, USA). All *P-*values were 2-sided, and a value of less than 0.05 was considered as having statistical significance.

## Results

### Lack of association between *SLC52A3* SNPs and ESCC susceptibility

The observed genotype frequencies for the two polymorphisms of *SLC52A3* in the controls conformed to the Hardy–Weinberg equilibrium (*P* = 0.06 and 0.80 for rs13042395 and rs3746803, respectively). No significant associations were observed between rs13042395 or SNP 3746803 and ESCC risk (*P* > 0.05, Table 2).

**Table 2:** Association between SNPs in *SLC52A3* and ESCC risk in the Chinese population (N_ESCC_ = 479, N_control_ = 479)

Genotype	ESCC	Control	OR (95 % CI)	*P*
	N (%)	N (%)		
rs13042395				
CC	135 (28.18)	147 (30.69)	1.00 (reference)	
CT	236 (49.27)	218 (45.51)	1.18 (0.88–1.59)	0.28
TT	108 (22.55)	114 (23.80)	1.03 (0.73–1.47)	0.86
CT + TT	344 (71.82)	332 (69.31)	1.14 (0.86–1.50)	0.36
CC + CT	371 (77.45)	365 (76.20)	1.00 (reference)	
TT	108 (22.55)	114 (23.80)	0.93 (0.69–1.26)	0.65
rs3746803				
GG	425 (88.73)	424 (88.52)	1.00 (reference)	
GA	55 (11.48)	53 (11.06)	1.00 (0.67–1.49)	0.99
AA	1 (0.21)	2 (0.42)	0.50 (0.05–5.52)	0.57
GA + AA	54 (11.27)	55 (11.48)	0.98 (0.66–1.46)	0.92
GG + GA	478 (99.79)	477 (99.58)	1.00 (reference)	
AA	1 (0.21)	2 (0.42)	0.50 (0.05–5.52)	0.57

All associations are significant at *P* < 0.05

*SNP* single nucleotide polymorphism, *ESCC* esophageal squamous cell carcinoma, *OR* odds ratio, *95 % CI* 95 % confidence interval

### Association of SNPs with ESCC tumor characteristics at the time of diagnosis

Significant associations were observed when we stratified the ESCC cases by tumor characteristics (Table 3). For rs13042395, TT genotype carriers were less likely to have regional lymph node metastasis (OR = 0.55, 95 % CI, 0.31–0.98) than CC genotype carriers. This meant the risk of having a higher degree of regional lymph node metastasis for TT genotype carriers was 0.55-fold less than that for CC genotype carriers in ESCC patients. No association was found between rs13042395 and other tumor characteristics such as tumor size, depth of tumor invasion, distant metastasis and TNM classification. For rs3746803, no associations were observed with ESCC tumor characteristics.

**Table 3:** Association between SNPs in *SLC52A3* and clinical characteristics of ESCC in the Chinese population (N_ESCC_ = 343)

Genotype	Tumor size (≤3 cm/4–5 cm/≥6 cm)		Depth of tumor invasion ((Tis + T1)/T2/T3/T4)		Regional lymph node metastasis (N0/N1/N2/N3)		Distant metastasis (M0/M1)		TNM classification ((0 + I)/II/III/IV)	
	OR (95 % CI)	*P*	OR (95 % CI)	*P*	OR (95 % CI)	*P*	OR (95 % CI)	*P*	OR (95 % CI)	*P*
rs13042395										
CC	1.00 (Reference)		1.00 (Reference)		1.00 (Reference)		1.00 (Reference)		1.00 (Reference)	
CT	1.17 (0.73–1.86)	0.53	0.90 (0.52–1.55)	0.71	0.83 (0.52–1.33)	0.45	0.83 (0.14–5.04)	0.84	0.79 (0.41–1.26)	0.33
TT	1.18 (0.67–2.06)	0.57	0.82 (0.43–1.56)	0.55	0.55 (0.31–0.98)	0.04	0.59 (0.05–6.63)	0.67	0.72 (0.49–1.27)	0.25
CT + TT	1.17 (0.75–1.82)	0.49	0.88 (0.52–1.46)	0.61	0.73 (0.47–1.14)	0.16	0.75 (0.14–4.17)	0.74	0.76 (0.49–1.20)	0.24
CC + CT	1.00 (Reference)		1.00 (Reference)		1.00 (Reference)		1.00 (Reference)		1.00 (Reference)	
TT	1.07 (0.67–1.70)	0.79	0.88 (0.52–1.50)	0.63	0.62 (0.38–1.01)	0.06	0.66 (0.08–5.77)	0.71	0.84 (0.52–1.34)	0.46
rs3746803										
GG	1.00 (Reference)		1.00 (Reference)		1.00 (Reference)		1.00 (Reference)		1.00 (Reference)	
GA + AA	1.1 (0.57–2.09)	0.78	1.4 (0.65–3.00)	0.39	1.06 (0.55–2.03)	0.87	1.73 (0.20–15.02)	0.62	1.3 (0.67–2.51)	0.43

All associations are significant at *P* < 0.05

*SNP* single nucleotide polymorphism, *ESCC* esophageal squamous cell carcinoma, *OR* odds ratio, *95 % CI* 95 % confidence interval

### Univariate and multivariate analysis of SNPs with ESCC relapse-free survival

**Fig. 1:**
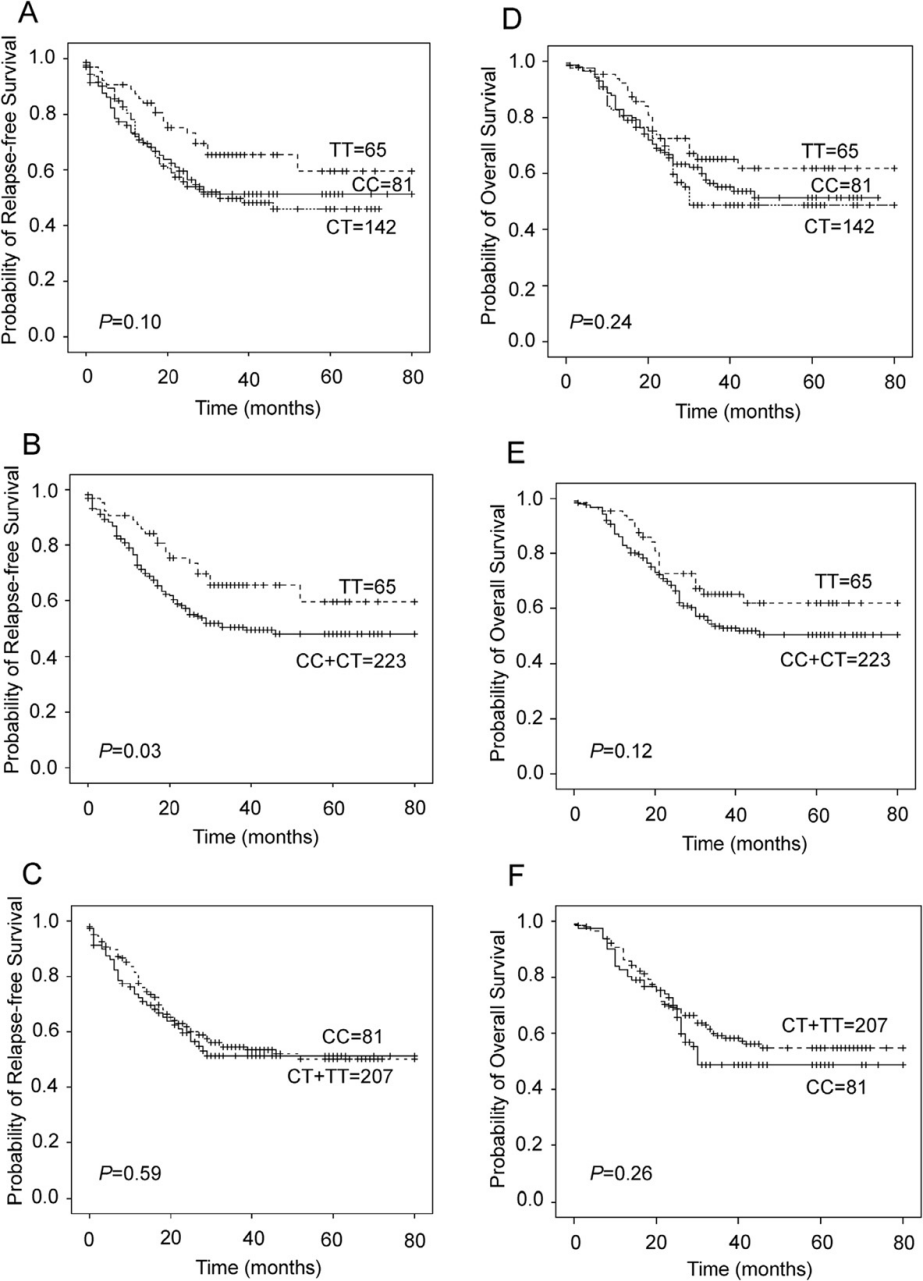
Kaplan-Meier analysis of relapse-free survival and overall survival for rs13042395 polymorphisms in the *SLC52A3* gene in ESCC cases. **a** The median relapse-free survival time for CC, CT and TT genotype carriers was 24, 23 and 30 months, respectively. The differences of relapse-free survival time among CC, CT and TT genotype carriers were not significant (*P* = 0.10). **b** The median relapse-free survival time for (CC + CT) genotype carriers and TT genotype carriers was 23 and 30 months, respectively. Relapse-free survival time of TT genotype carriers was significantly longer compared with that of (CC + CT) genotype carriers (*P* = 0.03). **c** The median relapse-free survival time for CC genotype carriers and (CT + TT) genotype carriers was 24 and 25 months, respectively. The difference of relapse-free survival time between CC and (CT + TT) genotype carriers was not significant (*P* = 0.59). **d** The median overall survival time for CC, CT and TT genotype carriers was 26, 27 and 32 months, respectively. The differences of overall survival time among CC, CT and TT genotype carriers were not significant (*P* = 0.24). **e** The median overall survival time for (CC + CT) genotype carriers and TT genotype carriers was 27 and 32 months, respectively. The difference of overall survival time between (CC + CT) and TT genotype carriers was not significant (*P* = 0.12). **f** The median overall survival time for CC genotype carriers and (CT + TT) genotype carriers was 26 and 29 months, respectively. The difference of overall survival time between CC and (CT + TT) genotype carriers was not significant (*P* = 0.26)

For rs13042395, the median relapse-free survival time for (CC + CT) genotype carriers and TT genotype carriers was 23 and 30 months, respectively. Relapse-free survival time of TT genotype carriers was significantly longer compared with that of (CC + CT) genotype carriers (*P* = 0.03) (Fig. 1b). According to univariate Cox regression analysis, TT genotype carriers had a decreased risk for relapse after surgery (HR = 0.60, 95 % CI, 0.38–0.96) compared with (CC + CT) genotype carriers (Table 4). This result suggests that the relative risk of relapse after surgery for TT genotype carriers is 0.60-fold less than that for the (CC + CT) genotype carriers in ESCC patients. Also, depth of tumor invasion, regional lymph node metastasis and TNM classification were risk factors for ESCC relapse-free survival with the *P*-value for trend (*P*_*trend*_) < 0.05. In multivariate Cox regression models, gender, age, rs13042395, rs3746803, tumor size, depth of tumor invasion, regional lymph node metastasis, tumor location, TNM classification, radiotherapy after surgery and chemotherapy after surgery were adjusted for each other. Multivariate analysis showed that rs13042395 (HR = 0.62, 95 % CI, 0.38–0.99 for TT *vs*. (CC + CT)), depth of tumor invasion, regional lymph node metastasis (HR = 2.06, 95 % CI, 1.36–3.13 for N1 *vs*. N0; HR = 2.88, 95 % CI, 1.70–4.86 for N2 *vs*. N0; HR = 2.08, 95 % CI, 1.01–4.30 for N3 *vs*. N0) and radiotherapy after surgery (HR = 1.68, 95 % CI, 1.16–2.43) were linked with ESCC relapse (Table 4). Cox regression analysis in 84 patients who had radiotherapy after surgery showed that TT genotype carriers were more likely to have relapse-free survival (HR = 0.46, 95 % CI, 0.21–0.98) compared with (CC + CT) genotype carriers (Additional file 1: Table S1). This indicates that the four factors were independent prognostic factors for ESCC relapse. However, rs3746803 was not shown to be associated with ESCC relapse-free survival either by univariate or multivariate analysis (Table 4, Additional file 2: Figure S1A).

**Table 4:** Univariate analyses and multivariate analysis of factors associated with relapse-free survival for ESCC patients (N_ESCC_ = 288)

Variables	HR	95 % CI for HR		*P*
		Lower	Upper	
Univariate analyses				
Gender				
Female	1.00	Reference		
Male	1.12	0.75	1.66	0.58
Age (years)	0.99	0.97	1.01	0.42
rs13042395				
CC	1.00	Reference		
CT	1.05	0.70	1.57	0.82
TT	0.62	0.36	1.06	0.08
CT + TT	0.90	0.61	1.33	0.60
CC + CT	1.00	Reference		
TT	0.60	0.38	0.96	0.03
rs3746803				
GG	1.00	Reference		
GA + AA	1.23	0.72	2.12	0.44
Tumor size (cm)				
≤3	1.00	Reference		
4–5	1.48	0.97	2.27	0.07
≥6	1.40	0.85	2.31	0.18
*P*_*trend*_				0.18
Depth of tumor invasion				
Tis + T1	1.00	Reference		
T2	5.42	1.21	24.24	0.03
T3	10.78	2.66	43.70	<0.001
T4	4.68	0.42	51.70	0.21
*P*_*trend*_				0.001
Regional lymph node metastasis				
N0	1.00	Reference		
N1	2.63	1.74	3.98	<0.001
N2	3.73	2.24	6.23	<0.001
N3	3.46	1.74	6.91	<0.001
*P*_*trend*_				<0.001
Tumor location				
Upper thoracic	1.00	Reference		
Middle thoracic	0.63	0.34	1.16	0.14
Lower thoracic	0.72	0.38	1.36	0.31
*P*_*trend*_				0.31
TNM classification				
0 + I	1.00	Reference		
II	5.83	1.81	18.76	0.003
III	14.21	4.47	45.17	<0.001
*P*_*trend*_				<0.001
Radiotherapy after surgery				
No	1.00	Reference		
Yes	2.12	1.48	3.03	<0.001
Chemotherapy after surgery				
No	1.00	Reference		
Yes	1.69	1.17	2.44	0.005
Multivariate analysis				
rs13042395				
CC + CT	1.00	Reference		
TT	0.62	0.38	0.99	0.046
Depth of tumor invasion				
Tis + T1	1.00	Reference		
T2	4.23	0.94	19.11	0.06
T3	6.37	1.54	26.40	0.01
T4	1.47	0.13	16.94	0.76
*P*_*trend*_				0.001
Regional lymph node metastasis				
N0	1.00	Reference		
N1	2.06	1.36	3.13	<0.001
N2	2.88	1.70	4.86	0.001
N3	2.08	1.01	4.30	<0.001
*P*_*trend*_				0.047
Radiotherapy after surgery				
No	1.00	Reference		
Yes	1.68	1.16	2.43	0.006

All associations are significant at *P* < 0.05

*ESCC* esophageal squamous cell carcinomas, *HR* hazard ratio, *95 % CI* 95 % confidence interval

### Univariate and multivariate analysis of SNPs with ESCC overall survival

According to Kaplan-Meier survival curve, the differences of overall survival among genotypes of rs13042395 were not significant (Fig. 1d–f). Also, the difference of overall survival between GG and (GA + AA) genotype of rs3746803 was not significant (Additional file 2: Figure S1B). Univariate Cox regression analysis showed that neither rs13042395 nor rs3746803 was associated with ESCC overall survival after surgery (Additional file 3: Table S2). In multivariate Cox regression analysis, significant association with ESCC overall survival was found for regional lymph node metastasis, TNM classification and radiotherapy after surgery, but not for rs13042395 or rs3746803 (Additional file 3: Table S2).

### Transcriptional activity of the rs13042395 in ESCC cells

**Fig. 2:**
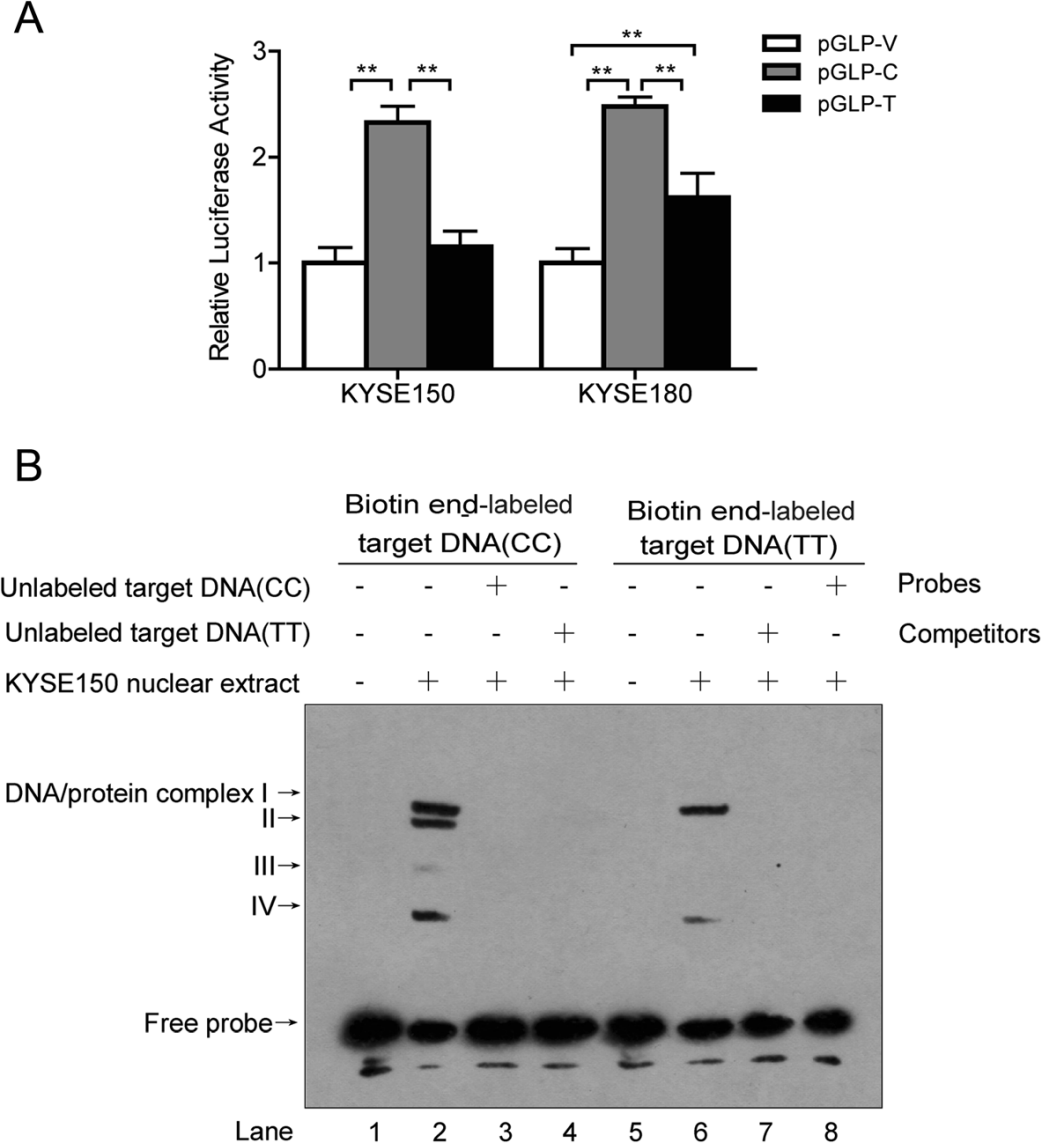
Promoter activity of rs13042395 polymorphisms in the *SLC52A3* gene in ESCC cells. **a** Luciferase reporter activity of the *SLC52A3* rs13042395 locus in KYSE150 and KYSE180 cells. Constructions of pGLP-V, pGLP-C or pGLP-T were co-transfected into the cells with pRL-TK in the indicated amounts. The empty vector pGLP-V was used as a control. *Firefly* luciferase activity was normalized to *Renilla* luciferase activity of the internal control. The experiments were repeated three times. Error bars indicate 95 % confidence intervals, one asterisk indicates statistical significance with *P* < 0.05, two asterisks indicate statistical significance with *P* < 0.01. Statistical significance was determined by two-sided one-way analysis of variance along with the Bonferroni post hoc test. **b** Electrophoretic mobility shift assay analysis of specific interaction between nuclear proteins and the rs13042395 site of *SLC52A3*. The nuclear protein was extracted from KYSE150 cells. Three micrograms of extract were incubated with a biotin end-labeled oligonucleotide GGCCAGTGCACCGTCATTGTGTGGGCTGGG (CC probes, *lanes 1 through 4*) or GGCCAGTGCACCGTTATTGTGTGGGCTGGG (TT probes, *lanes 5 through 8*) in the rs13042395 site. Binding specificity was confirmed by chasing labeled CC or TT probes with a 200-fold molar excess of unlabeled CC (*lane 4*) or TT probe (*lane 7*). Labeled probes free of nuclear extracts migrated as shown in lanes1 and 4. Four shift bands presented in lane 2 (CC polymorphism), but only two in lane 6 (TT polymorphism)

A dual luciferase reporter assay was conducted to investigate the transcriptional activity of rs13042395 in KYSE150 and KYSE180 cells (Fig. 2a). Empty vector pGLP-V was used as a control. The assay showed that both pGLP-C and pGLP-T had greater relative luciferase activity than the control vector when expressed in KYSE150 and KYSE180 cells. The increased range for pGLP-C relative luciferase activity was greatly elevated (*P* < 0.01), while that for the pGLP-T was not obvious. This suggests that the CC genotype of rs13042395 has stronger transcription activity for the down-stream gene (*SLC52A3*) and promotes *SLC52A3* expression.

### DNA-binding activity for CC genotype of rs13042395

In order to identify whether DNA-binding activity on rs13042395 plays an important role in *SLC52A3* overexpression, we used an EMSA assay to search for potential factors that could interact with rs13042395 (Fig. 2b). Nuclear extracts prepared from KYSE150 cells were incubated with biotin-labeled oligonucleotides for rs13042395 loci containing either the CC or TT genotype. This binding reaction generated four protein-DNA complexes (I, II, III and IV) between the CC genotype oligonucleotides and nuclear protein (shifted bands) (Fig. 2b, lane 2). To determine the specificity of the binding complex, we added a 200-fold molar excess of unlabeled oligonucleotide to the reaction. As a result, the shifted bands were completely ablated (Fig. 2b, lanes 3 and 8). Interestingly, neither protein-DNA complex II nor complex III was generated with the TT genotype oligonucleotide. Collectively, these results demonstrate that a specific interaction exists between nuclear proteins and the DNA sequence containing C allele rather than the T allele in the rs13042395 locus. These results are consistent with the above results demonstrating that the CC genotype of rs13042395 in humans promotes *SLC52A3* gene expression, probably via binding with additional transcription factors.

## Discussion

In recent years, the role of rs13042395 in ESCC susceptibility has been controversial. In 2010, rs13042395 was initially found to be related to ESCC by Wang et al. [8]. However, in later years, researchers found no association between this SNP and ESCC [17–20]. Our results are consistent with the latter. The present study shows that neither rs13042395 nor rs3746803 is related to ESCC risk. For rs13042395, TT genotype carriers were less likely to have regional lymph node metastasis (compared with CC genotype carriers) and more likely to have better relapse-free survival (compared with (CC + CT) genotype carriers). The CC genotype of rs13042395 likely promotes down-stream *SLC52A3* gene expression in human ESCC, probably by binding with specific transcription factors.

According to our study, rs13042395 in *SLC52A3* plays an important role in regional lymph node metastasis. This is supported by our demonstration that rs13042395 and regional lymph node metastasis status are independent prognostic factors for relapse-free survival. Moreover, we show that the CC genotype of rs13042395 could act by promoting *SLC52A3* expression by causing the binding of additional transcription factors. Previous studies demonstrated that regional lymph node metastasis is significantly associated with relapse-free survival and overall survival in ESCC patients [22, 23]. More lymph node metastasis leads to worse survival. Also, it has been demonstrated that the *SLC52A3* gene is overexpressed in ESCC cells, promotes ESCC cell proliferation and protects against cell death [14]. Increases in cancer cell proliferation and decreases in cancer cell death will inevitably promote cancer cell metastasis to lymph nodes and thereby result in poor survival in ESCC patients. Therefore, it is reasonable to conclude that the CC genotype of rs13042395 in humans leads to *SLC52A3* overexpression, which promotes ESCC cell proliferation and protects against cell death. This biologic function makes CC genotype carriers more likely to have lymph node metastasis and worse relapse-free survival. Conversely, TT genotype carriers are less likely to have lymph node metastasis and better relapse-free survival, as observed in present study. All evidence indicates that rs13042395 plays an essential role in ESCC prognosis and is a potential predictive marker for progression and prognosis of ESCC.

RFT2 is a crucial transporter, encoded by the *SLC52A3* gene, involved in epithelial cell uptake of riboflavin for nutritional utilization [12, 13]. Riboflavin deficiency has been identified as a risk factor for ESCC in high-risk areas [24–27]. Prior research indicates that dietary riboflavin could ameliorate the effects of ESCC carcinogens [28]. Large population intervention trials also suggest that riboflavin supplementation can reduce the incidence of esophageal cancer [29–32]. However, in the ESCC patients supplemented with riboflavin, blood riboflavin in 34.2 % of the subjects was still lower than normal, despite sufficient dietary riboflavin [32]. Our study shows that the CC genotype of rs13042395 can promote *SLC52A3* expression. A recent study indicates that, in ESCC, high levels of riboflavin intake via *SLC52A3* overexpression promotes tumorigenesis by sustaining cell proliferation and protecting against cell death [14]. Taking this one step further, there might exist a self-balancing regulation system in which *SLC52A3* could suppress itself when overexpressed in humans. As a result, dysfunction of the *SLC52A3* gene could occur, and the ability of RFT2 to transport dietary riboflavin becomes inhibited. This may partly explain why blood riboflavin is deficient, even though the dietary riboflavin was sufficient.

Regarding rs3746803, we found rs3746803 had no association with susceptibility, tumor characteristics and survival of ESCC patients, consistent with a previous study [15]. This could be because the MAF of rs3746803 is too low in the Chaoshan population.

There are some limitations in the present study. First, our study is a retrospective study, which may lead to statistical bias in the analyses. Replicating studies with larger sample size and with a prospective design is necessary to clarify the association between rs13042395 and rs3746803 and ESCC. Second, our study indicates that the CC genotype of rs13042395 promotes *SLC52A3* expression, probably via binding with specific transcription factors that generated complexes II and III. However, the identity of the transcription factors, their underlying biological functions, and the mechanisms by which they regulate *SLC52A3* expression all remain unknown. All of these scientific issues require further research.

## Conclusions

The present study demonstrates that rs13042395 polymorphisms in the *SLC52A3* gene play an important role in regional lymph node metastasis and relapse-free survival of ESCC patients. The rs13042395 could enhance *SLC52A3* expression in humans, and this enhancement is probably due to additional transcription factor binding. These results might provide clues for health care planning and clinical research of ESCC, increasing important areas with the application of targeted therapies.

### Abbreviations

CI, confidence interval; EAC, esophageal adenocarcinoma; EC, esophageal cancer; EMSA, electrophoretic mobility shift assay; ESCC, esophageal squamous cell carcinoma; GWAS, genome-wide association studies; HR, hazard ratio; MAF, minor allele frequency; OR, odds ratio; PCR, polymerase chain reaction; *P*_*trend*_, P value for trend; RFT2, human riboflavin transporter 2; SNPs, single nucleotide polymorphisms.

## Additional files

Additional file 1: Table S1. Relapse-free survival for 84 ESCC patients who had radiotherapy after surgery. (DOC 34 kb) Additional file 2: Figure S1. Kaplan-Meier analysis of relapse-free survival and overall survival for the rs3746803 loci of *SLC52A3* in 288 esophageal squamous cell carcinoma cases. (**A**) The median relapse-free survival time for GG genotype carriers and (GA + AA) genotype carriers were 25 months and 20 months, respectively. The difference of relapse-free survival time between GG genotype carriers and (GA + AA) genotype carriers was not significant (*P* = 0.46). (**B**) The median overall survival time for GG genotype carriers and (GA + AA) genotype carriers were 28 months and 27 months, respectively. The difference of overall survival time between GG genotype carriers and (GA + AA) genotype carriers was not significant (*P* = 0.92). (TIF 1240 kb) Additional file 3: Table S2. Univariate analyses and multivariate analysis of factors associated with overall survival for ESCC patients (N_ESCC_ = 288). (DOC 107 kb)
