# Supplementary material for: Single nucleotide polymorphism rs13042395 in the SLC52A3 gene as a biomarker for regional lymph node metastasis and relapse-free survival of esophageal squamous cell carcinoma patients
Source: BMC Cancer. 2016 Jul 29;16:560. doi: 10.1186/s12885-016-2588-3 (PMC4966773; doi:10.1186/s12885-016-2588-3)
Supplement: Additional file 1: Table S1. — Relapse-free survival for 84 ESCC patients who had radiotherapy after surgery. (DOC 34 kb) [file 12885_2016_2588_MOESM1_ESM.doc]

| **Table S1.** Relapse-free survival for 84 ESCC patients who had radiotherapy after surgery. | | | | |
| --- | --- | --- | --- | --- |
| Variables | HR^†^ | 95% CI for HR | | *P* |
|  |  | Lower | Upper |  |
| rs13042395 |  |  |  |  |
| CC | 1.00 | Reference | |  |
| CT | 1.00 | 0.55 | 1.82 | 0.99 |
| TT | 0.46 | 0.20 | 1.06 | 0.07 |
| CT+TT | 1.39 | 0.79 | 0.45 | 0.42 |
| CC+CT | 1.00 | Reference | |  |
| TT | 0.46 | 0.21 | 0.98 | **0.04** |
| ESCC, esophageal squamous cell carcinoma; HR, hazard ratio; 95% CI, 95% confidence interval. | | | | |
| All associations are significant at *P*<0.05. | | | | |
| ^†^ Adjusted for age and sex. | | | | |
