# Supplementary figures and images for: Single nucleotide polymorphism rs13042395 in the SLC52A3 gene as a biomarker for regional lymph node metastasis and relapse-free survival of esophageal squamous cell carcinoma patients
Source: BMC Cancer. 2016 Jul 29;16:560. doi: 10.1186/s12885-016-2588-3 (PMC4966773; doi:10.1186/s12885-016-2588-3)

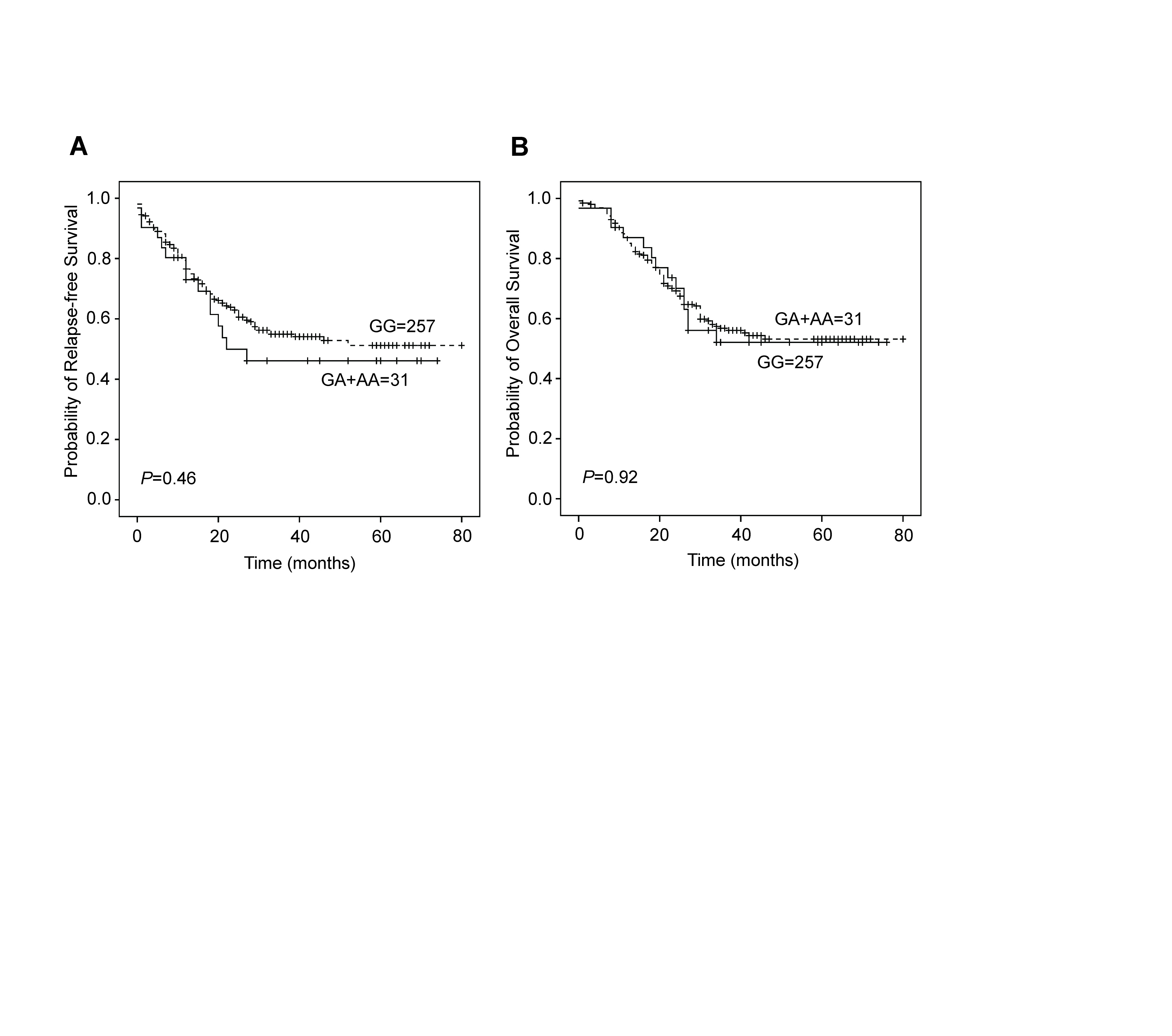

Supplement: Additional file 2: Figure S1. — Kaplan-Meier analysis of relapse-free survival and overall survival for the rs3746803 loci of SLC52A3 in 288 esophageal squamous cell carcinoma cases. (A) The median relapse-free survival time for GG genotype carriers and (GA + AA) genotype carriers were 25 months and 20 months, respectively. The difference of relapse-free survival time between GG genotype carriers and (GA + AA) genotype carriers was not significant (P = 0.46). (B) The median overall survival time for GG genotype carriers and (GA + AA) genotype carriers were 28 months and 27 months, respectively. The difference of overall survival time between GG genotype carriers and (GA + AA) genotype carriers was not significant (P = 0.92). (TIF 1240 kb) [file 12885_2016_2588_MOESM2_ESM.tif]
