# Supplementary material for: Single nucleotide polymorphism rs13042395 in the SLC52A3 gene as a biomarker for regional lymph node metastasis and relapse-free survival of esophageal squamous cell carcinoma patients
Source: BMC Cancer. 2016 Jul 29;16:560. doi: 10.1186/s12885-016-2588-3 (PMC4966773; doi:10.1186/s12885-016-2588-3)
Supplement: Additional file 3: Table S2. — Univariate analyses and multivariate analysis of factors associated with overall survival for ESCC patients (NESCC = 288). (DOC 107 kb) [file 12885_2016_2588_MOESM3_ESM.doc]

| **Table S2.** Univariate and multivariate analyses of factors associated with overall survival for ESCC patients (N_ESCC_=288). | | | | |
| --- | --- | --- | --- | --- |
| Variables | HR | 95% CI for HR | | *P* |
|  |  | Lower | Upper |  |
| **Univariate analyses** |  |  |  |  |
| Gender |  |  |  |  |
| Female | 1.00 | Reference | |  |
| Male | 1.15 | 0.77 | 1.74 | 0.50 |
| Age (years) | 1.01 | 0.99 | 1.03 | 0.21 |
| rs13042395 |  |  |  |  |
| CC | 1.00 | Reference | |  |
| CT | 0.88 | 0.59 | 1.33 | 0.56 |
| TT | 0.64 | 0.38 | 1.09 | 0.10 |
| CT+TT | 0.80 | 0.55 | 1.18 | 0.27 |
| CC+CT | 1.00 | Reference | |  |
| TT | 0.70 | 0.44 | 1.11 | 0.12 |
| rs3746803 |  |  |  |  |
| GG | 1.00 | Reference | |  |
| GA+AA | 1.06 | 0.60 | 1.84 | 0.85 |
| Tumor size (cm) |  |  |  |  |
| ≤3 | 1.00 | Reference | |  |
| 4﹣5 | 1.71 | 1.07 | 2.73 | 0.02 |
| ≥6 | 2.43 | 1.47 | 4.02 | <0.001 |
| *P_trend_* |  |  |  | 0.002 |
| Depth of tumor invasion |  |  |  |  |
| Tis+T1 | 1.00 | Reference | |  |
| T2 | 5.09 | 1.14 | 22.74 | 0.03 |
| T3 | 9.31 | 2.30 | 37.76 | 0.002 |
| T4 | 15.85 | 2.64 | 95.27 | 0.003 |
| *P_trend_* |  |  |  | 0.003 |
| Regional lymph node metastasis | |  |  |  |
| N0 | 1.00 | Reference | |  |
| N1 | 3.39 | 2.17 | 5.30 | <0.001 |
| N2 | 6.11 | 3.63 | 10.27 | <0.001 |
| N3 | 5.30 | 2.72 | 10.33 | <0.001 |
| *P_trend_* |  |  |  | <0.001 |
| Tumor location |  |  |  |  |
| Upper thoracic | 1.00 | Reference | |  |
| Middle thoracic | 0.79 | 0.39 | 1.60 | 0.51 |
| Lower thoracic | 1.34 | 0.66 | 2.70 | 0.42 |
| TNM classification |  |  |  |  |
| 0+I | 1.00 | Reference | |  |
| II | 4.43 | 1.36 | 14.38 | 0.01 |
| III | 13.72 | 4.32 | 43.55 | <0.001 |
| *P_trend_* |  |  |  | <0.001 |
| Radiotherapy after surgery | |  |  |  |
| No | 1.00 | Reference | |  |
| Yes | 0.96 | 0.65 | 1.41 | 0.82 |
| Chemotherapy after surgery | |  |  |  |
| No | 1.00 | Reference | |  |
| Yes | 1.21 | 0.82 | 1.79 | 0.33 |
| **Multivariate analysis** |  |  |  |  |
| Regional lymph node metastasis | |  |  |  |
| N0 | 1.00 | Reference | |  |
| N1 | 3.72 | 2.35 | 5.88 | <0.001 |
| N2 | 6.36 | 3.66 | 11.04 | <0.001 |
| N3 | 7.05 | 3.37 | 14.77 | <0.001 |
| *P_trend_* |  |  |  | <0.001 |
| Tumor size (cm) |  |  |  |  |
| ≤3 | 1.00 | Reference | |  |
| 4﹣5 | 1.03 | 0.63 | 1.68 | 0.92 |
| ≥6 | 1.67 | 0.99 | 2.84 | 0.06 |
| *P_trend_* |  |  |  | 0.05 |
| Tumor location |  |  |  |  |
| Upper thoracic | 1.00 | Reference | |  |
| Middle thoracic | 0.46 | 0.22 | 0.97 | 0.04 |
| Lower thoracic | 0.81 | 0.39 | 1.69 | 0.58 |
| *P_trend_* |  |  |  | 0.007 |
| Radiotherapy after surgery | |  |  |  |
| No | 1.00 | Reference | |  |
| Yes | 0.59 | 0.38 | 0.90 | 0.02 |
| ESCC, esophageal squamous cell carcinoma; HR, hazard ratio; 95% CI, 95% confidence interval. | | | | |
| All associations are significant at *P*<0.05. | | | | |
